# Antiplatelets or anticoagulants? Secondary prevention in cervical artery dissection: an updated meta-analysis

**DOI:** 10.1186/s42466-022-00188-7

**Published:** 2022-06-13

**Authors:** Ei Zune The, Ne Naing Lin, Ching Jocelyn Chan, Jason Cher Wei Loon, Benjamin Yong-Qiang Tan, Chee Seong Raymond Seet, Hock Luen Teoh, Joy Vijayan, Leong Litt Leonard Yeo

**Affiliations:** 1 Division of Neurology, Department of Medicine, National University Hospital, Singapore, Singapore; 2 University Hospitals of Leicester NHS Trust, Leicester, UK; 3 Leicester General Hospital, Gwendolen Rd, Leicester, LE5 4PW UK; 4 University of Medicine 1, Yangon, Myanmar; 5 National University of Singapore, Singapore, Singapore

**Keywords:** Internal carotid artery, Vertebral artery, Extracranial artery dissection, Aspirin, Anticoagulants, Secondary prevention, Meta-analysis, Stroke

## Abstract

**Background:**

Extracranial artery dissection involving either internal carotid artery or vertebral artery is a major cause of stroke in adults under 50 years of age. There is no conclusive evidence whether antiplatelets or anticoagulants are better suited in the treatment of extracranial artery dissection.

**Objectives:**

To determine whether antiplatelets or anticoagulants have advantage over the other in the treatment of extracranial artery dissection for secondary prevention of recurrent ischemic events or death.

**Methods:**

Present meta-analysis followed Preferred Reporting Items for Systematic reviews and Meta-Analyses (PRISMA) 2020 statement. Database search was done in Medline, Cochrane Central Register of Controlled Trials (CENTRAL) and ClinicalTrials.gov from inception to May 2021 using pre-defined search strategy. Additional studies were identified from reference lists from included studies, reviews and previous meta-analyses. Outcome measures were ischaemic stroke, ischaemic stroke or transient ischaemic attack (TIA), and death.

**Results:**

Two RCTs and 64 observational studies were included in the meta-analysis. While the outcome measures of stroke, stroke or TIA and death were numerically higher with antiplatelet use, there were no statistically significant differences between antiplatelets and anticoagulants.

**Conclusion:**

We found no significant difference between antiplatelet and anticoagulation treatment after extracranial artery dissection. The choice of treatment should be tailored to individual cases.

**Supplementary Information:**

The online version contains supplementary material available at 10.1186/s42466-022-00188-7.

## Background

Cervical artery dissection is an important cause of stroke in patients under 50 years of age [1, 2]. It involves dissection of either internal carotid artery or vertebral artery or both and can be unilateral or bilateral or multi-vessel [3]. It is estimated to affect 2.6 to 2.9 per 100,000 individuals per year [4]. While it can resolve spontaneously within 3 to 6 months, it can recur in a minority of individuals and mortality has been reported up to 5% of the affected individuals [4].

In severe cases, especially in multi-vessel dissections, interventional treatment with stenting may be needed. Conversely, in milder cases, conservative treatment with medication and regular follow-up till spontaneous resolution is indicated [5]. However, the choice of medication, in the form of antiplatelet or anticoagulant agents, is still largely dependent on the treating physicians’ preference and evidence to support one treatment over the other is lacking. Recent randomized controlled trials (RCT) have been limited in sample size [6, 7] and meta-analyses aggregating the data have not been conclusive [8–13].

It is the aim of the present study to include recent clinical trials to update the data and determine whether antiplatelets or anticoagulants have advantage over the other in the treatment of extracranial artery dissection for the secondary prevention of ischaemic events or death.

## Methods

Present meta-analysis followed Preferred Reporting Items for Systematic Reviews and Meta-analysis (PRISMA) 2020 guidelines [14].

### Data search

An electronic database search was made in MEDLINE database and CENTRAL and ClinicalTrials.gov from inception to May 2021. The search words used and the steps involved for MEDLINE database search is shown in table S1 [Please see the details in Additional file 1] and it was adapted for searches in CENTRAL and ClinicalTrials.gov. Titles and abstracts from search results were scrutinized to determine the eligibility of a result to be included in the analysis. Additional search was made by reviewing references from previous meta-analyses and review papers. Subsequently, selected studies were then read in details for data extraction.

Screening, study selection and data extraction were done by a team of two investigators (CJC and JCWL) and independently reviewed by another team of two investigators (EZT and NNL). Any disagreement on study inclusion and data extraction were resolved by discussion. Summary of methods involved is shown in Table 1.

**Table 1:** PICOs framework and applied inclusion and exclusion criteria

PICOs	Inclusion Criteria	Exclusion Criteria
Population	Adults with extracranial internal carotid or vertebral artery dissections (spontaneous or due to recreational activities or sport related minor injuries)	Cases with severe traumatic causes of arterial dissections Intracranial dissections Children
Intervention vs Comparison	Antiplatelet treatment (single or dual) including aspirin, indobufen, dipyridamole, ticlopidine, clopidogrel, sulfinpyrazone Anticoagulant treatment (traditional or newer agents) including heparin, coumarin, warfarin, dabigatran, rivaroxaban, apixaban	Stents or Surgical repairs as first treatment
Outcome	Ischaemic stroke Ischaemic stroke or TIA Death	
Study design	Randomized controlled trials (RCT) Controlled clinical trials (CCT) Non-randomized studies including observational studies and cases series Must provide evidence of dissection Outcome data allows comparison between antiplatelets and anticoagulants Databases: Medline, CENTRAL, ClinicalTrials.gov Search period: Inception to May 2021	Systematic review Meta-analysis Case overlaps Less than five cases

PICOs = Population, Intervention, Comparison, Outcome and study design, TIA = transient ischaemic attack, CENTRAL = Cochrane Central Register of Controlled Trials

The above table (Table 1) with its legend should appear at the end of the Data Search sub-section and before Inclusion and Exclusion Criteria sub-section under METHODS section

### Inclusion and exclusion criteria

Studies are included in the analysis if it fulfills following inclusion criteria—1. The study must provide evidence of dissection by either magnetic resonance imaging (MRI) or magnetic resonance angiography or computed tomography (CT) angiography or digital subtraction angiography. 2. The outcome data allows comparison between patients on antiplatelets and anticoagulants. A study is excluded—1. If there are four or less cases, 2. If cases with severe traumatic causes of arterial dissections, for example, motor vehicle collision, could not be excluded. Dissection associated with minor trauma, for example, recreational activities or sport related minor injuries are allowed to be included in the meta-analysis. 3. If concurrent intracranial dissection could not be excluded, 4. If cases treated with stents or surgical repairs as first treatment could not be excluded, and 5. If the study population comprises of children.

### Outcome measures

Outcome measures for analysis include death related to carotid or vertebral artery dissection, ischaemic stroke and a composite outcome of stroke or TIA. If a study is to be included in the analysis, it must report at least one of the outcome measures.

### Data extraction

Patients with surgical treatment or switching from one treatment group to another or receiving both antiplatelets and anticoagulants were excluded. In circumstances of studies with overlapping populations, the study with the most complete data or larger sample population was selected. Patients were grouped as either receiving antiplatelet or anticoagulation, most times using vitamin-K-antagonists, depending on the initial treatment they received. In cases with initial treatment with heparin, it is classified as in anticoagulant group if it is prolonged and used anticoagulation dose and it is classified as in antiplatelet group if it is given only for the initial days before transitioning to antiplatelet treatment. Data extraction was done with the aim to get as much complete data as possible, i.e. per-protocol data as much as possible.

### Data analysis

Data analyses were done using Review Manager (version 5.4) developed by The Cochrane Collaboration [15]. Risk difference (RD) with 95% confidence interval (95% CI) was used since it was expected that there will be zero outcome events and Odd Ratio (OR) or Risk Ratio (RR) could not be calculated for each study before pooling the data in the meta-analysis. Nevertheless, ORs based on total population and outcome events were calculated for each outcome measure.

## Results

**Fig. 1:**
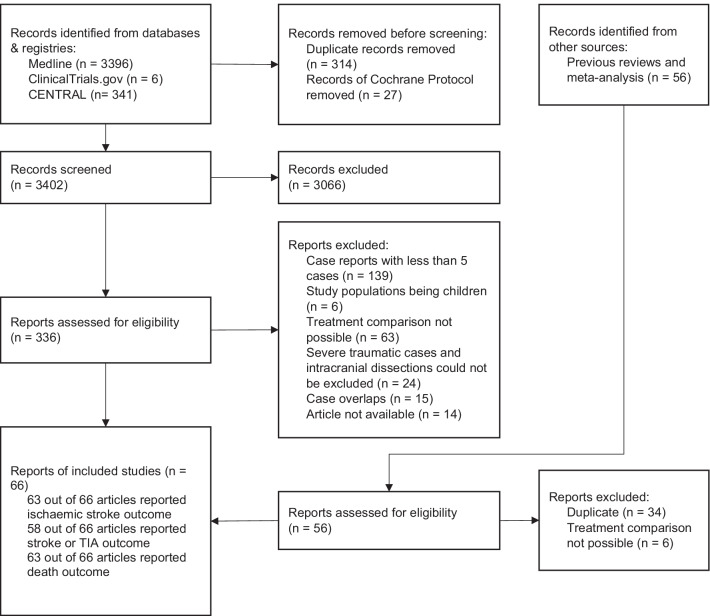
Study flow chart

The database and registries searches were done on 22nd June 2021 and the search was limited to end of May 2021 from the inception of the database and the registries. The search identified 3402 results after removing duplicates. After initial screening of titles and abstracts, 336 articles were selected for full reading. Subsequently, 286 articles were excluded with reasons of less than 5 cases (139), study population being children (6), review articles (25), treatment comparison not possible (63), severe traumatic cases and intracranial dissections could not be excluded (24), population overlaps (15) and article not available (14). As a result, 50 articles were available to be included in the analysis [6, 7, 11, 16–62]. Additional searches done from the reference list of the included articles identified a further 16 articles and were added to the analysis [63–78]. The steps involved in article selection process is shown in Fig. 1.

There are only two RCTs [6, 7]—Cervical Artery Dissection in Stroke Study (CADISS) and Biomarkers and Antithrombotic Treatment in Cervical Artery Dissection (TREAT–CAD) – and the rest is made up of mostly observation studies in the form of case reports, case series, and diagnostic studies. There are 25 studies [16–19, 21–24, 27, 31, 32, 35, 46, 47, 50, 61, 63–69, 71, 73] reporting outcomes on internal carotid artery dissection only, 13 studies [20, 26, 28–30, 33, 34, 42, 52, 56, 57, 62, 70] reporting outcomes on vertebral artery dissection only and 28 studies [6, 7, 11, 25, 36–41, 43–45, 48, 49, 51, 53–55, 58–60, 72, 74–78] reporting outcomes on both. There were two studies [41, 52] with overlapping cases of vertebral artery dissection and data is taken from the study [52] published later in time. And the data on carotid artery dissection is taken from the other study [41] published earlier in time since it included both types of dissections. The follow-up period in each studies varies from less than 1 month to more than 3 months: 49 studies [6, 7, 11, 17–20, 22, 24, 25, 27, 29–31, 33–35, 38–52, 54, 57, 59, 63–66, 68–70, 72–78] with follow-up period of 3 or more months and 17 studies [16, 21, 23, 26, 28, 32, 36, 37, 53, 55, 56, 58, 60–62, 67, 71] with follow-up period of less than 3 months.

For CADISS RCT, data is taken from the results [10] published in 2015 and not the results [79] published in 2019. The reason for this is that there are many individuals who are not still on the initial treatment given at the start of randomization and it could not be certain that the effects of that treatment continue to exist. In TREAT-CAD RCT, there are clinical outcomes as well as MRI surrogate findings [11] and the MRI findings of acute ischaemic lesions without clinical symptoms have been taken as ischaemic stroke.

### Ischaemic stroke

**Fig. 2:**
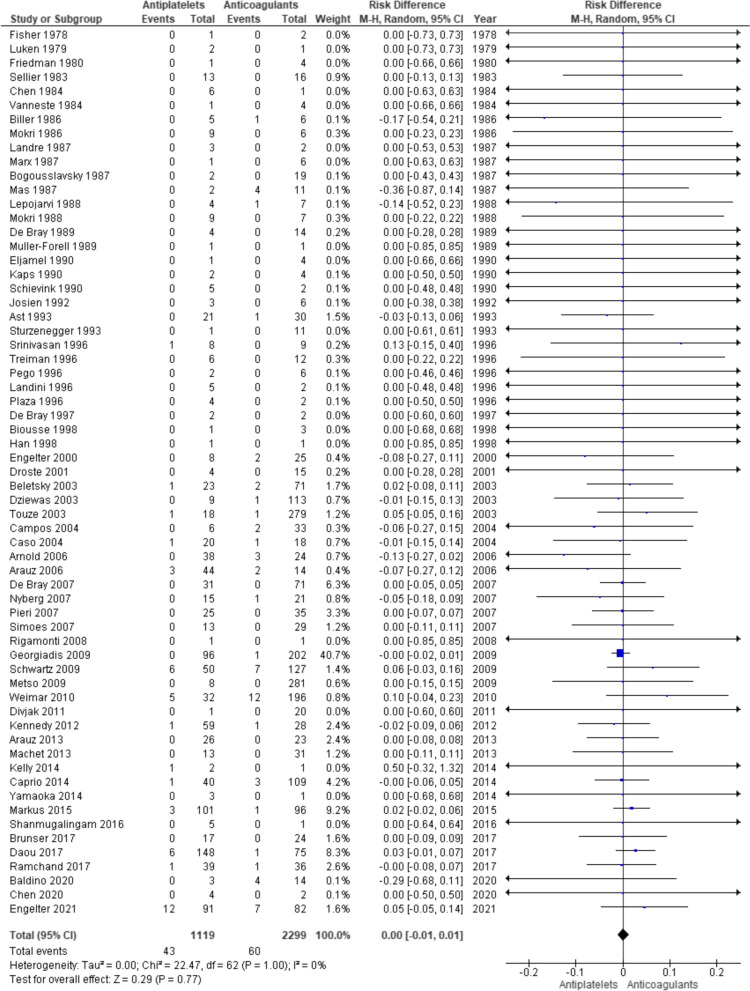
Forest plot for ischaemic stroke outcome

There are 63 studies included in the analysis for the outcome of ischaemic stroke [6, 7, 11, 16–36, 38–50, 52–77]. There are 3418 individuals in total and 1119 individuals received antiplatelets and 2299 individual received anticoagulants. There are 43 (3.84%) and 60 (2.61%) ischaemic stroke outcomes in antiplatelet group and anticoagulant group, respectively (OR 1.49). The risk difference was not statistically significant (0.00 [ − 0.01, 0.01], p = 0.77). Random effect model was used and there was no significant heterogeneity (I^2^ = 0%, p = 1.00) (Fig. 2).

### Stroke or TIA

**Fig. 3:**
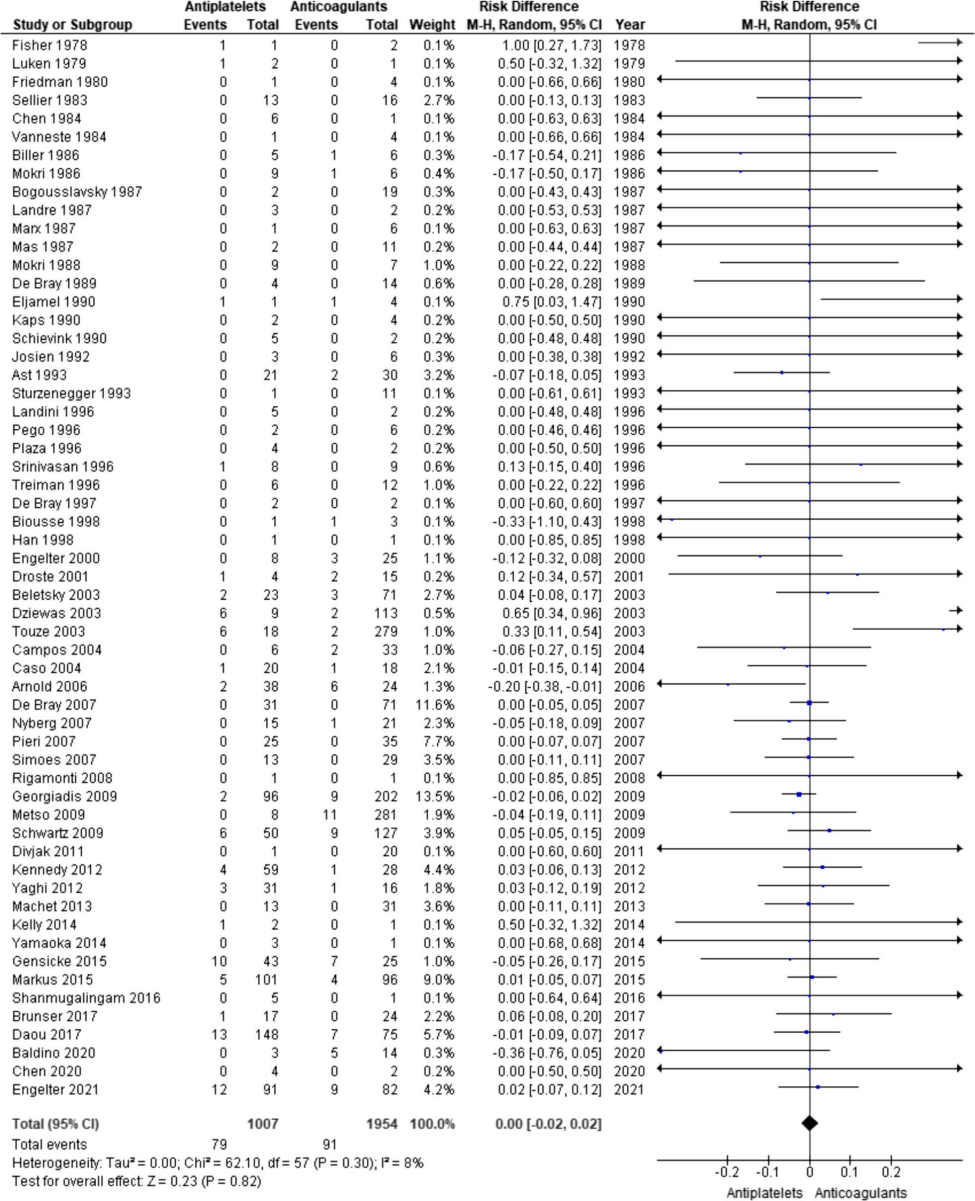
Forest plot for ischaemic stroke or TIA outcome

There are 58 studies included in the analysis for the outcome of stroke or TIA [6, 7, 11, 16–20, 22, 24–36, 38–40, 42–48, 50, 51, 53, 55–59, 61–78]. There are 2961 individuals in total and 1007 individuals received antiplatelets and 1954 individual received anticoagulants. There are 79 (7.85%) and 91 (4.66%) stroke or TIA outcomes in antiplatelet group and anticoagulant group, respectively (OR 1.74). The risk difference was not statistically significant (0.00 [ − 0.02, 0.02], p = 0.82). Random effect model was used and there was no significant heterogeneity (I^2^ = 8%, p = 0.30) (Fig. 3).

### Death

**Fig. 4:**
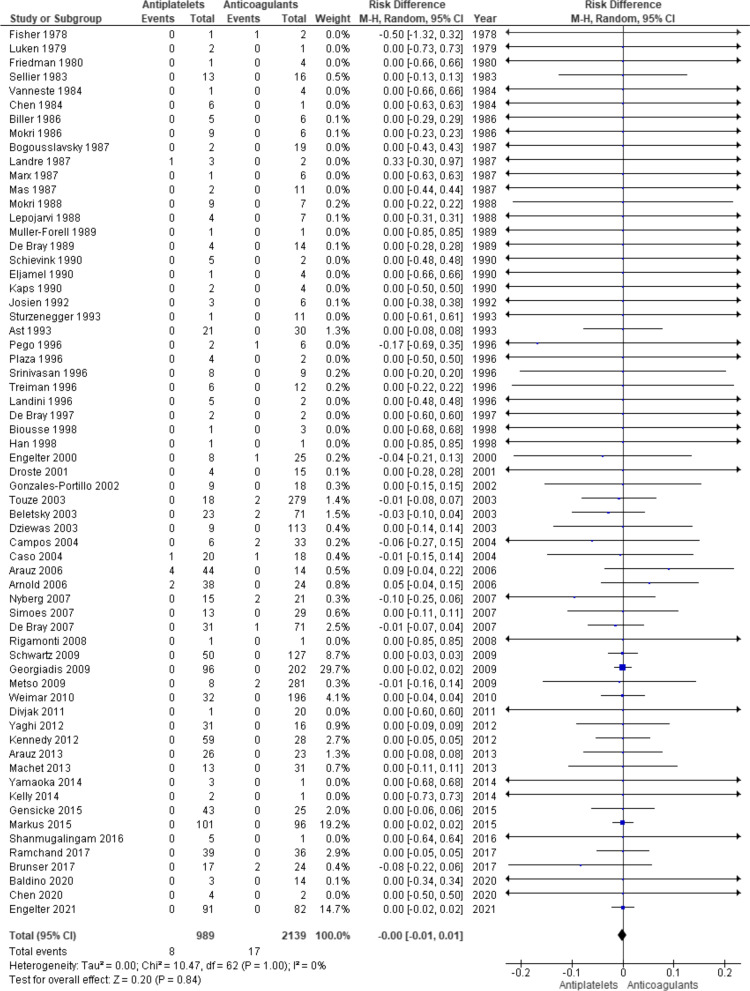
Forest plot for death outcome

There are 63 studies included in the analysis for the death outcome [6, 7, 11, 16–44, 46–53, 55–58, 60–78]. There are 3128 individuals in total and 989 individuals received antiplatelets and 2139 individuals received anticoagulants. There are 8 (0.81%) and 17 (0.79%) deaths in antiplatelet group and anticoagulant group, respectively (OR 1.02). The risk difference was not statistically significant (0.00 [ − 0.01, 0.01], p = 0.84). Random effect model was used and there was no significant heterogeneity (I^2^ = 0%, p = 1.00) (Fig. 4).

### Subgroup with RCT data alone

**Fig. 5:**
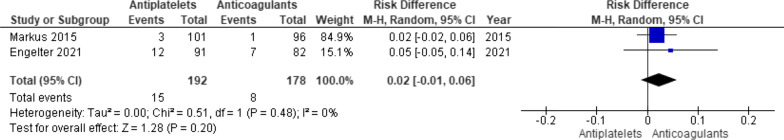
Forest plot for ischaemic stroke outcome for RCTs

**Fig. 6:**
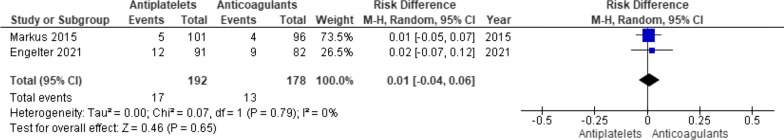
Forest plot for stroke or TIA outcome for RCTs

Analyses with data from the only two RCTs on this topic were performed. For ischaemic stroke outcome, there are higher numbers of ischaemic stroke events in antiplatelet group compared to anticoagulant group (7.81% vs. 4.49%, respectively; OR 1.80, p = 0.20), but this did not reach significance (Fig. 5). Similarly, for the combined stroke and TIA outcome, there are higher number of events in antiplatelet group than in anticoagulant group (8.85% vs. 7.30%, respectively; OR 1.23, p = 0.65) but this did not reach significance (Fig. 6). No deaths were reported in both RCTs.

### Subgroup analyses with carotid artery or vertebral artery dissection alone

Analyses with data on carotid artery dissection alone and vertebral artery dissection alone were performed as well. There are no risk difference to minimal risk difference with no statistical significance in the analyses for carotid artery dissection alone: in favour of antiplatelet treatment for ischaemic stroke outcome (total 1403 subjects in 36 studies; − 0.01 [ − 0.02, 0.01], p = 0.52), no risk difference for ischaemic stroke or TIA outcome (total 1030 subjects in 29 studies; 0.00 [ − 0.05, 0.05], p = 0.93), and no risk difference for death outcome (total 1347 subjects in 36 studies; 0.00 [ − 0.01, 0.01], p = 0.91). [Please see the details in figure S1 to S3 in Additional file 1.]

Similarly, there are minimal risk differences with no statistical significance in the analyses for vertebral artery dissection alone: in favour of antiplatelet treatment for ischaemic stroke outcome (total 555 subjects in 19 studies; − 0.02 [ − 0.06, 0.02], p = 0.26), in favour of antiplatelet treatment for ischaemic stroke or TIA outcome (total 237 subjects in 15 studies; − 0.02 [ − 0.08, 0.04], p = 0.50), and in favour of anticoagulant treatment for death outcome (total 448 subjects in 19 studies; 0.01 [ − 0.04, 0.05], p = 0.76). [Please see the details in Additional file 1: Figs. S4 to S6]

## Discussion

In the literature, there have been five meta-analyses that compared the results of using antiplatelets and anticoagulants in the secondary prevention of cervical artery dissection [8, 10–13]. Despite the various methodologies used, there is still no conclusive evidence that either class of the antithrombotic medication is superior to the other. Present meta-analysis with updated data also failed to find statistically significant differences between the two treatments (Figs. 2, 3, 4). However, it differs from the rest in that there are two RCTs, CADISS and TREAT-CAD [6, 7], included in the present meta-analysis. Both of these RCTs included both internal carotid and vertebral artery dissections and each failed to show significant differences between the two treatments. This is in agreement with the results of the present meta-analyses overall or with data from just these two RCTs (Figs. 5, 6) or with data on carotid and vertebral artery dissections separately (Additional file 1: Figs. S1 to S6).

CADISS was the first RCT to be published with the aim to determine the feasibility of a clinical trial to compare the effects of antiplatelets and anticoagulants in cervical artery dissection [80]. However, it found no statistically significant differences between the two treatments (in both per-protocol analysis and intention-to-treat analysis). Nonetheless, it highlighted that the diagnostic imaging criteria of dissection were often not applied correctly in clinical practice [6]. With the realization of lower than expected clinical outcome rates in CADISS and the RCT being underpowered, another RCT, TREAT-CAD, attempted to overcome this by adding MRI surrogate outcomes to determine non-inferiority of antiplatelets to anticoagulation [7]. However, TREAT-CAD found no significant differences between antiplatelets and anticoagulants nor non-inferiority of aspirin even after adding MRI findings, in both per-protocol and intention-to-treat analyses and despite a generous 12% non-inferiority margin.

CADISS also made power calculation using their findings on composite outcome of stroke, death or major bleeding (2.97%, 95% CI 0.62–8.44 with antiplatelets vs 2.08%, 95% CI 0.25–7.32 with anticoagulants) in per-protocol data to assess the feasibility of another trial. A sample size of 4876 individuals in each arm will be required for a study with 0.8 power and 0.05 significance level [6]. Such a trial would be too resource intensive and would take a tremendous amount of time to complete. As a comparison, it took over seven years to recruit 250 subjects (in total and around 200 per-protocol subjects) in CADISS (UK alone) and over five years to recruit 194 subjects (in total and 173 per-protocol subjects) in TREAT–CAD (Switzerland, Germany, and Denmark).

Comparing effects of antiplatelets and anticoagulants in medical treatment of extracranial artery dissection without taking into consideration of initial presentation, type of dissection (aneurysmal or stenotic or occlusive) and other demographic characteristics may be an oversimplification of a complex picture. Nevertheless, treatment with antithrombotics appears to be effective regardless of the underlying characteristics and may improve the survival of individuals with internal carotid or vertebral artery dissections. Rosati et al.reported that individuals on either antiplatelet treatment or anticoagulant treatment have significantly lowered risk of adverse outcomes [Hazard Ratio (HR) 0.15, 95% CI: 0.04–0.55, *p* = 0.005 and HR 0.19, 95% CI: 0.04–0.88, *p* = 0.034, respectively] compared to those without either treatment [81].

Baseline differences in types of dissections could have introduced some biases and obscured actual difference between the two treatments, i.e. type II error. In fact, selection bias was looked at by Ramchand et al. where he determined that there was a significantly higher degree of stenosis in individuals on anticoagulants and a non-significantly higher chance of receiving anticoagulants by patients with “stroke or TIA.”[60] The latter point was a significant finding in Daou et al. in which patients who received antiplatelet treatment have the lowest chance of presenting with stroke compared to those who received anticoagulation or combined treatments [59]. Another one factor that increased the difficulty in finding differences between anticoagulation and antiplatelet treatment was due to the fact that heparin is commonly used in the initial phase of treatment in addition to an antiplatelet, and this (early anticoagulation) was recommended in the early versions of European guidelines on management of stroke [82, 83].

Evidence on direct oral anticoagulants (DOAC) are still limited with very small sample size studies [54, 84, 85]. Similarly, the evidence for dual antiplatelet treatment is limited. In CADISS, almost half of the participants (28% received aspirin and clopidogrel, and 16% received aspirin and dipyridamole) received dual antiplatelet treatment. But the outcomes reported did not differentiate between single and dual antiplatelet treatments. In total (per-protocol), there were 3% ischaemic stroke outcome, 5% any stroke or TIA outcome and 0% major bleeding outcome. In contrast, in TREAT–CAD, only single antiplatelet treatment was used (Aspirin oral 300 mg or intravenous 250 mg). There were 8% ischaemic stroke, 0% TIA and 0% major bleeding (per-protocol sample). It is plausible that these differences in outcomes between these two RCTs could reflect the effects of single and dual antiplatelet treatments. However, more evidence is needed to either confirm or refute this.

Given the findings from CADISS and TREAT–CAD, there has been some consideration that the evidence to support antiplatelet treatment is weak albeit sufficient for the treatment of individuals with only symptoms and without haemodynamic compromise. Nevertheless, early treatment with either modality has been suggested, based on the finding that diffusion-weighted imaging (DWI) lesions have been detected to occur soon after diagnosis of the dissection [78].

## Limitations

Present meta-analysis considered only three outcomes which did not include bleeding adverse effect which is a potentially problematic adverse effect associated with antithrombotics. Also, cases due to severe trauma are excluded which could be considered as valid clinical variant that needs equal clinical attention. Cases that were stented and surgically treated were excluded since they could potentially be different from medically treated patients and could introduce further bias into the analysis. Present meta-analysis included studies with follow up periods less than three months and these could have altered the actual rates of outcome events and the final results. Majority of the included studies are observational studies which are prone to different biases. Per-protocol data was favored over intention-to-treat data which might be a pragmatic choice but can introduce bias. 

## Conclusion

Present meta-analysis did not find significant differences between antiplatelet and anticoagulant treatments despite increased sample size. The choice of antithrombotics should be tailored to the patient on an individual basis.

## Supplementary Information

**Additional file1:** **Table S1.** Search words used and the steps involved for MEDLINE database search. **Figure S1.** Forest plot for ischaemic stroke outcome in carotid artery dissection alone. **Figure S2.** Forest plot for ischaemic stroke or TIA outcome in carotid artery dissection alone. **Figure S3.** Forest plot for death outcome in carotid artery dissection alone. **Figure S4**. Forest plot for ischaemic stroke outcome in vertebral artery dissection alone. **Figure S5**. Forest plot for ischaemic stroke or TIA outcome in vertebral artery dissection alone. **Figure S6**. Forest plot for death outcome in vertebral artery dissection alone.

## Data Availability

Not applicable.

## Abbreviations

CADISS: Cervical artery dissection in stroke study
CCT: Controlled clinical trials
CENTRAL: Cochrane central register of controlled trials
CI,: Confidence interval
CT: Computed tomography
DOAC: Direct oral anticoagulants
DWI: Diffusion-weighted imaging
HR: Hazard ratio
MRI: Magnetic resonance imaging
OR: Odd ratio
PICOs: Population, intervention, comparison, outcome and study design
PRISMA: Preferred reporting items for systematic reviews and meta-analyses
RCT: Randomized controlled trials
RD: Risk difference
RR: Risk ratio
TIA: Transient ischaemic attack
TREAT–CAD: Biomarkers and antithrombotic treatment in cervical artery dissection
