# Supplementary material for: Antiplatelets or anticoagulants? Secondary prevention in cervical artery dissection: an updated meta-analysis
Source: Neurol Res Pract. 2022 Jun 13;4:23. doi: 10.1186/s42466-022-00188-7 (PMC9190132; doi:10.1186/s42466-022-00188-7)

Table S1. Search words used and the steps involved for MEDLINE database search

Step	Search words
1	(("carotid artery injuries"[MeSH Terms]) OR ("carotid artery, internal, dissection"[MeSH Terms])) OR ("vertebral artery dissection"[MeSH Terms])
2	((carotid injur*) OR (carotid dissecti*)) OR (carotid trauma)
3	((vertebral injur*) OR (vertebral dissecti*)) OR (vertebral trauma)
4	1 OR 2 OR 3
5	((carotid arteries[MeSH Terms]) OR (carotid artery disease[MeSH Terms])) OR (carotid artery thrombosis[MeSH Terms])
6	(("vertebral artery"[All Fields]) OR ("vertebral artery disease"[All Fields])) OR ("vertebral artery thrombosis"[All Fields])
7	5 OR 6
8	((aneurysm, dissecting[MeSH Terms]) OR (aneurysm, false[MeSH Terms])) OR (aneurysm, ruptured[MeSH Terms])
9	"wounds, nonpenetrating"[MeSH Terms]
10	((traumatic dissection*) OR ("traumatic aneurysm"[All Fields])) OR ("traumatic pseudoaneurysm"[All Fields])
11	(blunt injur*) OR ("blunt trauma"[All Fields])
12	"dissecting aneurysm"[All Fields]
13	("rupture, spontaneous"[MeSH Terms]) OR ("rupture"[MeSH Terms])
14	"spontaneous dissection"[All Fields]
15	8 OR 9 OR 10 OR 11 OR 12 OR 13 OR 14
16	7 AND 15
17	4 OR 16
18	"platelet aggregation inhibitors"[MeSH Terms]
19	("blood platelets"[MeSH Terms]) OR ("platelet aggregation"[MeSH Terms])
20	"fibrinolytic agents"[MeSH Terms]
21	"anticoagulants"[MeSH Terms]
22	"thrombolytic therapy"[MeSH Terms]
23	"thromboembolism"[MeSH Terms]
24	"thrombosis"[MeSH Terms]
25	((antiplatelet*[Text Word]) OR (antithromb*[Text Word])) OR (anticoagula*[Text Word])
26	((((((aspirin[Text Word]) OR (acetylsalicyl*[Text Word])) OR (indobufen[Text Word])) OR (dipyridamole[Text Word])) OR (ticlopidine[Text Word])) OR (clopidogrel[Text Word])) OR (sulfinpyrazone[Text Word])) OR (sulphinpyrazone[Text Word])
27	(((heparin*[Text Word]) OR (coumar*[Text Word])) OR (coumadin[Text Word])) OR (warfarin[Text Word])
28	18 OR 19 OR 20 OR 21 OR 22 OR 23 OR 24 OR 25 OR 26 OR 27
29	17 AND 28
30	applied “Humans” filter in species

Figure S1. Forest plot for ischaemic stroke outcome in carotid artery dissection alone

Study or Subgroup	Antiplatelets Events	Antiplatelets Total	Anticoagulants Events	Anticoagulants Total	Weight	Risk Difference M-H, Random, 95% CI	Year
Fisher 1978	0	1	0	2	0.1%	0.00 [-0.73, 0.73]	1978
Luken 1979	0	2	0	1	0.1%	0.00 [-0.73, 0.73]	1979
Friedman 1980	0	1	0	4	0.1%	0.00 [-0.66, 0.66]	1980
Sellier 1983	0	13	0	16	1.7%	0.00 [-0.13, 0.13]	1983
Chen 1984	0	6	0	1	0.1%	0.00 [-0.63, 0.63]	1984
Vanneste 1984	0	1	0	4	0.1%	0.00 [-0.66, 0.66]	1984
Biller 1986	0	5	1	6	0.2%	-0.17 [-0.54, 0.21]	1986
Mokri 1986	0	9	0	6	0.5%	0.00 [-0.23, 0.23]	1986
Landre 1987	0	3	0	2	0.1%	0.00 [-0.53, 0.53]	1987
Bogousslavsky 1987	0	2	0	19	0.1%	0.00 [-0.43, 0.43]	1987
Mas 1987	0	2	4	11	0.1%	-0.36 [-0.87, 0.14]	1987
Lepojarvi 1988	0	4	1	7	0.2%	-0.14 [-0.52, 0.23]	1988
Muller-Forell 1989	0	1	0	1	0.0%	0.00 [-0.85, 0.85]	1989
De Bray 1989	0	4	0	14	0.4%	0.00 [-0.28, 0.28]	1989
Eljamel 1990	0	1	0	4	0.1%	0.00 [-0.66, 0.66]	1990
Kaps 1990	0	2	0	4	0.1%	0.00 [-0.50, 0.50]	1990
Schievink 1990	0	5	0	1	0.1%	0.00 [-0.64, 0.64]	1990
Ast 1993	0	21	1	30	2.9%	-0.03 [-0.13, 0.06]	1993
Srinivasan 1996	1	8	0	9	0.4%	0.13 [-0.15, 0.40]	1996
Treiman 1996	0	6	0	12	0.6%	0.00 [-0.22, 0.22]	1996
Biousse 1998	0	1	0	3	0.1%	0.00 [-0.68, 0.68]	1998
Engelter 2000	0	8	2	25	0.8%	-0.08 [-0.27, 0.11]	2000
Droste 2001	0	4	0	13	0.3%	0.00 [-0.28, 0.28]	2001
Dziewas 2003	0	7	0	71	1.0%	0.00 [-0.17, 0.17]	2003
Touze 2003	1	18	1	279	2.4%	0.05 [-0.05, 0.16]	2003
Caso 2004	1	10	1	9	0.4%	-0.01 [-0.29, 0.27]	2004
Arauz 2006	3	44	2	14	0.7%	-0.07 [-0.27, 0.12]	2006
Pieri 2007	0	10	0	14	1.2%	0.00 [-0.15, 0.15]	2007
Rigamonti 2008	0	1	0	1	0.0%	0.00 [-0.85, 0.85]	2008
Georgiadis 2009	0	96	1	202	80.0%	-0.00 [-0.02, 0.01]	2009
Metso 2009	1	4	3	140	0.2%	0.23 [-0.20, 0.65]	2009
Divjak 2011	0	1	0	20	0.1%	0.00 [-0.60, 0.60]	2011
Kennedy 2012	1	34	1	16	1.6%	-0.03 [-0.16, 0.10]	2012
Machet 2013	0	10	0	25	1.5%	0.00 [-0.13, 0.13]	2013
Caprio 2014	1	18	1	37	2.0%	0.03 [-0.09, 0.15]	2014
Baldino 2020	0	3	4	14	0.2%	-0.29 [-0.68, 0.11]	2020
**Total (95% CI)**		**366**		**1037**	**100.0%**	**-0.01 [-0.02, 0.01]**	
Total events	9		23				

Heterogeneity: Tau² = 0.00; Chi² = 10.31, df = 35 (P = 1.00); I² = 0%

Test for overall effect: Z = 0.65 (P = 0.52)

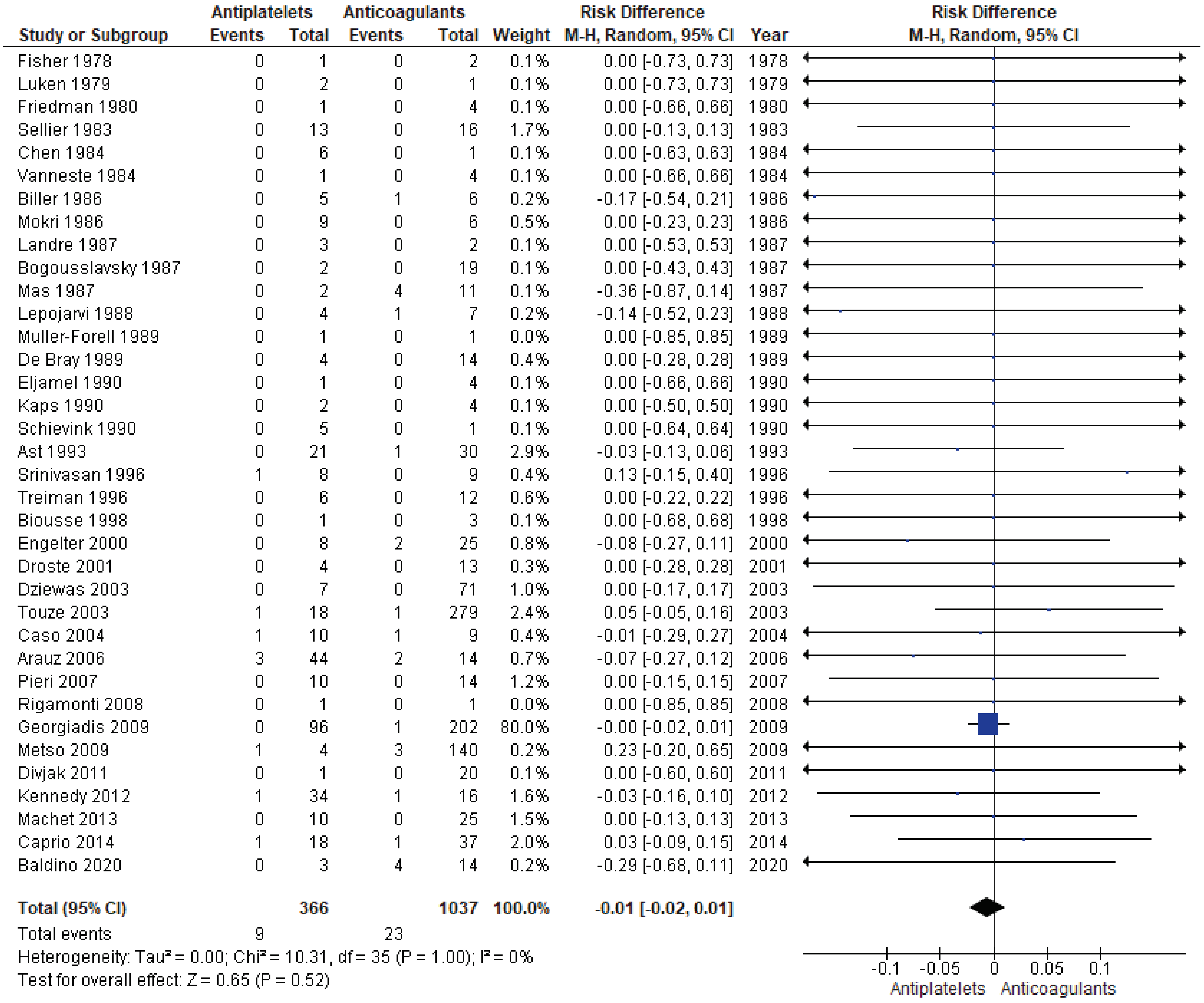

Figure S2. Forest plot for ischaemic stroke or TIA outcome in carotid artery dissection alone

Study or Subgroup	Antiplatelets Events	Antiplatelets Total	Anticoagulants Events	Anticoagulants Total	Weight	Risk Difference M-H, Random, 95% CI	Year
Fisher 1978	1	1	0	2	0.5%	1.00 [0.27, 1.73]	1978
Luken 1979	1	2	0	1	0.4%	0.50 [-0.32, 1.32]	1979
Friedman 1980	0	1	0	4	0.6%	0.00 [-0.66, 0.66]	1980
Sellier 1983	0	13	0	16	9.6%	0.00 [-0.13, 0.13]	1983
Chen 1984	0	6	0	1	0.6%	0.00 [-0.63, 0.63]	1984
Vanneste 1984	0	1	0	4	0.6%	0.00 [-0.66, 0.66]	1984
Mokri 1986	0	9	1	6	2.2%	-0.17 [-0.50, 0.17]	1986
Biller 1986	0	5	1	6	1.7%	-0.17 [-0.54, 0.21]	1986
Bogousslavsky 1987	0	2	0	19	1.4%	0.00 [-0.43, 0.43]	1987
Landre 1987	0	3	0	2	0.9%	0.00 [-0.53, 0.53]	1987
Marx 1987	0	1	0	6	0.6%	0.00 [-0.63, 0.63]	1987
De Bray 1989	0	4	0	14	3.0%	0.00 [-0.28, 0.28]	1989
Schievink 1990	0	5	0	1	0.6%	0.00 [-0.64, 0.64]	1990
Eljamel 1990	1	1	1	4	0.5%	0.75 [0.03, 1.47]	1990
Kaps 1990	0	2	0	4	1.0%	0.00 [-0.50, 0.50]	1990
Ast 1993	0	21	2	30	10.8%	-0.07 [-0.18, 0.05]	1993
Srinivasan 1996	1	8	0	9	3.0%	0.13 [-0.15, 0.40]	1996
Treiman 1996	0	6	0	12	4.5%	0.00 [-0.22, 0.22]	1996
Biousse 1998	0	1	1	3	0.4%	-0.33 [-1.10, 0.43]	1998
Engelter 2000	0	8	3	25	5.2%	-0.12 [-0.32, 0.08]	2000
Droste 2001	1	4	2	13	1.1%	0.10 [-0.37, 0.56]	2001
Touze 2003	6	18	2	279	4.5%	0.33 [0.11, 0.54]	2003
Pieri 2007	0	10	0	14	7.6%	0.00 [-0.15, 0.15]	2007
Rigamonti 2008	0	1	0	1	0.4%	0.00 [-0.85, 0.85]	2008
Georgiadis 2009	2	96	9	202	20.2%	-0.02 [-0.06, 0.02]	2009
Divjak 2011	0	1	0	20	0.7%	0.00 [-0.60, 0.60]	2011
Kennedy 2012	4	34	1	16	7.1%	0.06 [-0.11, 0.22]	2012
Machet 2013	0	10	0	25	9.0%	0.00 [-0.13, 0.13]	2013
Baldino 2020	0	3	5	14	1.5%	-0.36 [-0.76, 0.05]	2020
**Total (95% CI)**		**277**		**753**	**100.0%**	**0.00 [-0.05, 0.05]**	
Total events	17		28				

Heterogeneity: Tau² = 0.00; Chi² = 35.62, df = 28 (P = 0.15); I² = 21%

Test for overall effect: Z = 0.08 (P = 0.93)

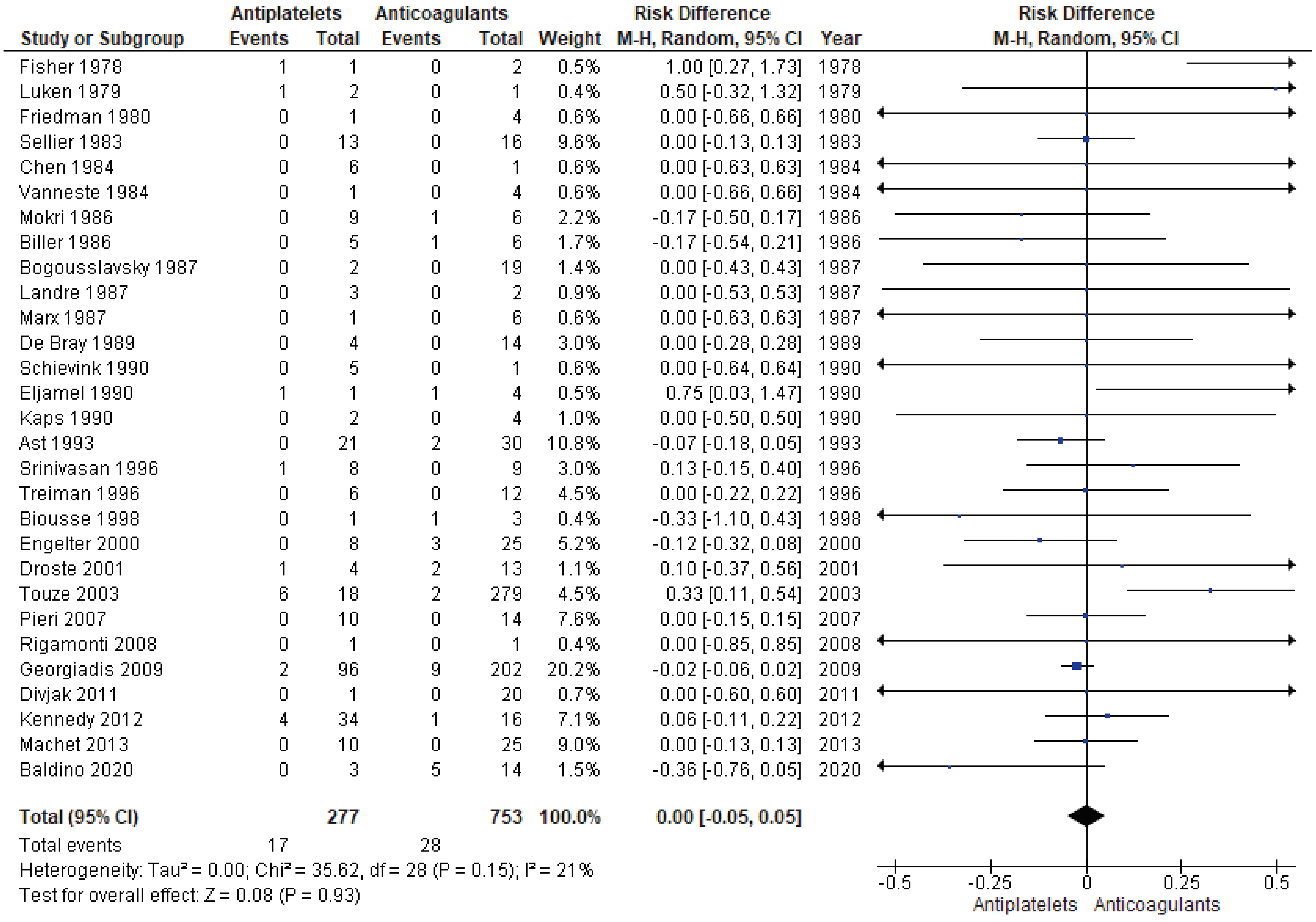

Figure S3. Forest plot for death outcome in carotid artery dissection alone

Study or Subgroup	Antiplatelets Events	Antiplatelets Total	Anticoagulants Events	Anticoagulants Total	Weight	Risk Difference M-H, Random, 95% CI	Year
Fisher 1978	0	1	1	2	0.0%	-0.50 [-1.32, 0.32]	1978
Luken 1979	0	2	0	1	0.0%	0.00 [-0.73, 0.73]	1979
Friedman 1980	0	1	0	4	0.0%	0.00 [-0.66, 0.66]	1980
Sellier 1983	0	13	0	16	1.2%	0.00 [-0.13, 0.13]	1983
Vanneste 1984	0	1	0	4	0.0%	0.00 [-0.66, 0.66]	1984
Chen 1984	0	6	0	1	0.0%	0.00 [-0.63, 0.63]	1984
Biller 1986	0	5	0	6	0.2%	0.00 [-0.29, 0.29]	1986
Mokri 1986	0	9	0	6	0.4%	0.00 [-0.23, 0.23]	1986
Bogousslavsky 1987	0	2	0	19	0.1%	0.00 [-0.43, 0.43]	1987
Landre 1987	1	3	0	2	0.0%	0.33 [-0.30, 0.97]	1987
Marx 1987	0	1	0	6	0.0%	0.00 [-0.63, 0.63]	1987
Lepojarvi 1988	0	4	0	7	0.2%	0.00 [-0.31, 0.31]	1988
Muller-Forell 1989	0	1	0	1	0.0%	0.00 [-0.85, 0.85]	1989
De Bray 1989	0	4	0	14	0.3%	0.00 [-0.28, 0.28]	1989
Schievink 1990	0	5	0	1	0.0%	0.00 [-0.64, 0.64]	1990
Eljamel 1990	0	1	0	4	0.0%	0.00 [-0.66, 0.66]	1990
Kaps 1990	0	2	0	4	0.1%	0.00 [-0.50, 0.50]	1990
Ast 1993	0	21	0	30	3.4%	0.00 [-0.08, 0.08]	1993
Srinivasan 1996	0	8	0	9	0.5%	0.00 [-0.20, 0.20]	1996
Treiman 1996	0	6	0	12	0.4%	0.00 [-0.22, 0.22]	1996
Biousse 1998	0	1	0	3	0.0%	0.00 [-0.68, 0.68]	1998
Engelter 2000	0	8	1	25	0.7%	-0.04 [-0.21, 0.13]	2000
Droste 2001	0	4	0	13	0.3%	0.00 [-0.28, 0.28]	2001
Gonzales-Portillo 2002	0	5	0	14	0.3%	0.00 [-0.24, 0.24]	2002
Dziewas 2003	0	7	1	71	0.7%	-0.01 [-0.19, 0.16]	2003
Touze 2003	0	18	2	279	3.7%	-0.01 [-0.08, 0.07]	2003
Caso 2004	1	10	0	9	0.3%	0.10 [-0.14, 0.34]	2004
Arauz 2006	4	44	0	14	1.2%	0.09 [-0.04, 0.22]	2006
Rigamonti 2008	0	1	0	1	0.0%	0.00 [-0.85, 0.85]	2008
Georgiadis 2009	0	96	0	202	79.4%	0.00 [-0.02, 0.02]	2009
Metso 2009	0	4	1	140	0.3%	-0.01 [-0.27, 0.26]	2009
Divjak 2011	0	1	0	20	0.1%	0.00 [-0.60, 0.60]	2011
Kennedy 2012	0	34	0	16	2.5%	0.00 [-0.09, 0.09]	2012
Machet 2013	0	10	0	25	1.1%	0.00 [-0.13, 0.13]	2013
Gensicke 2015	0	20	0	17	2.0%	0.00 [-0.10, 0.10]	2015
Baldino 2020	0	3	0	14	0.2%	0.00 [-0.34, 0.34]	2020
**Total (95% CI)**		**362**		**1012**	**100.0%**	**0.00 [-0.01, 0.01]**	
Total events	6		6				

Heterogeneity: Tau² = 0.00; Chi² = 5.71, df = 35 (P = 1.00); I² = 0%
Test for overall effect: Z = 0.12 (P = 0.91)

Risk Difference
M-H, Random, 95% CI

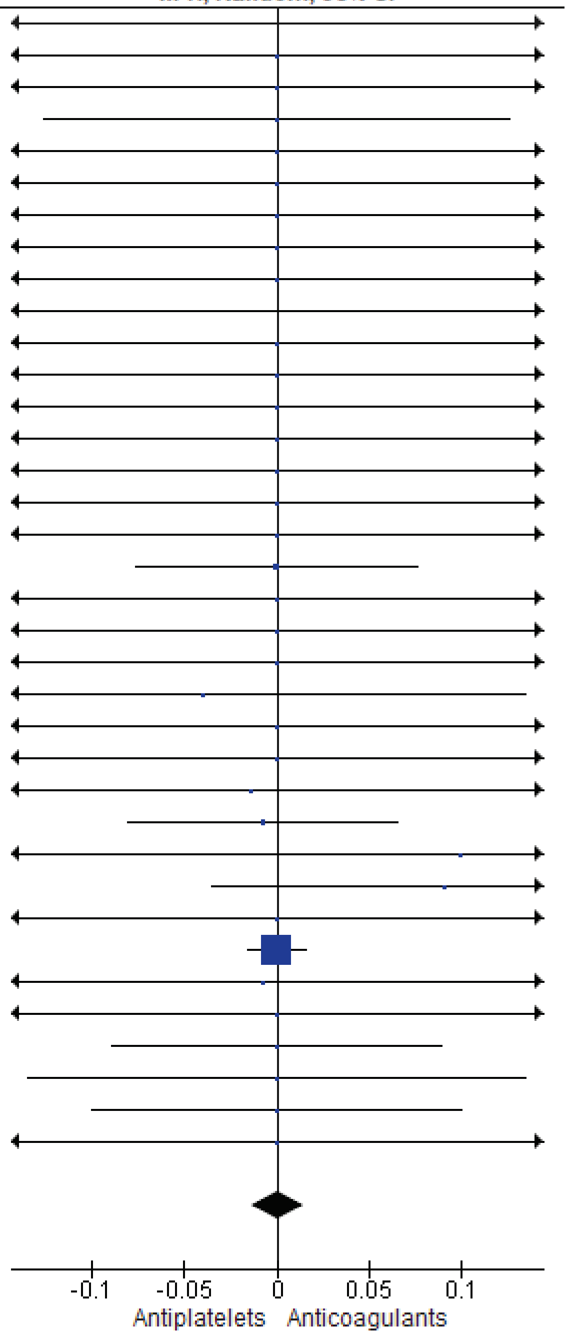

Figure S4. Forest plot for ischaemic stroke outcome in vertebral artery dissection alone

Study or Subgroup	Antiplatelets Events	Antiplatelets Total	Anticoagulants Events	Anticoagulants Total	Weight	Risk Difference M-H, Random, 95% CI	Year
Mas 1987	0	2	4	11	0.5%	-0.36 [-0.87, 0.14]	1987
Mokri 1988	0	9	0	7	2.9%	0.00 [-0.22, 0.22]	1988
Josien 1992	0	3	0	6	1.0%	0.00 [-0.38, 0.38]	1992
Sturzenegger 1993	0	1	0	11	0.4%	0.00 [-0.61, 0.61]	1993
Pego 1996	0	2	0	6	0.6%	0.00 [-0.46, 0.46]	1996
Plaza 1996	0	4	0	2	0.5%	0.00 [-0.50, 0.50]	1996
De Bray 1997	0	2	0	2	0.4%	0.00 [-0.60, 0.60]	1997
Han 1998	0	1	0	1	0.2%	0.00 [-0.85, 0.85]	1998
Caso 2004	0	10	1	9	2.1%	-0.11 [-0.36, 0.14]	2004
Arnold 2006	0	38	3	24	6.8%	-0.13 [-0.27, 0.02]	2006
Pieri 2007	0	15	0	21	12.1%	0.00 [-0.11, 0.11]	2007
Metso 2009	0	4	0	141	2.0%	0.00 [-0.26, 0.26]	2009
Kennedy 2012	0	26	0	13	11.2%	0.00 [-0.11, 0.11]	2012
Arauz 2013	0	26	0	23	23.1%	0.00 [-0.08, 0.08]	2013
Machet 2013	0	4	0	10	1.6%	0.00 [-0.29, 0.29]	2013
Caprio 2014	0	26	2	79	33.4%	-0.03 [-0.09, 0.04]	2014
Yamaoka 2014	0	3	0	1	0.3%	0.00 [-0.68, 0.68]	2014
Shanmugalingam 2016	0	5	0	1	0.3%	0.00 [-0.64, 0.64]	2016
Chen 2020	0	4	0	2	0.5%	0.00 [-0.50, 0.50]	2020
**Total (95% CI)**		**185**		**370**	**100.0%**	**-0.02 [-0.06, 0.02]**	
Total events	0		10				

Heterogeneity: Tau² = 0.00; Chi² = 5.91, df = 18 (P = 1.00); I² = 0%
Test for overall effect: Z = 1.14 (P = 0.26)

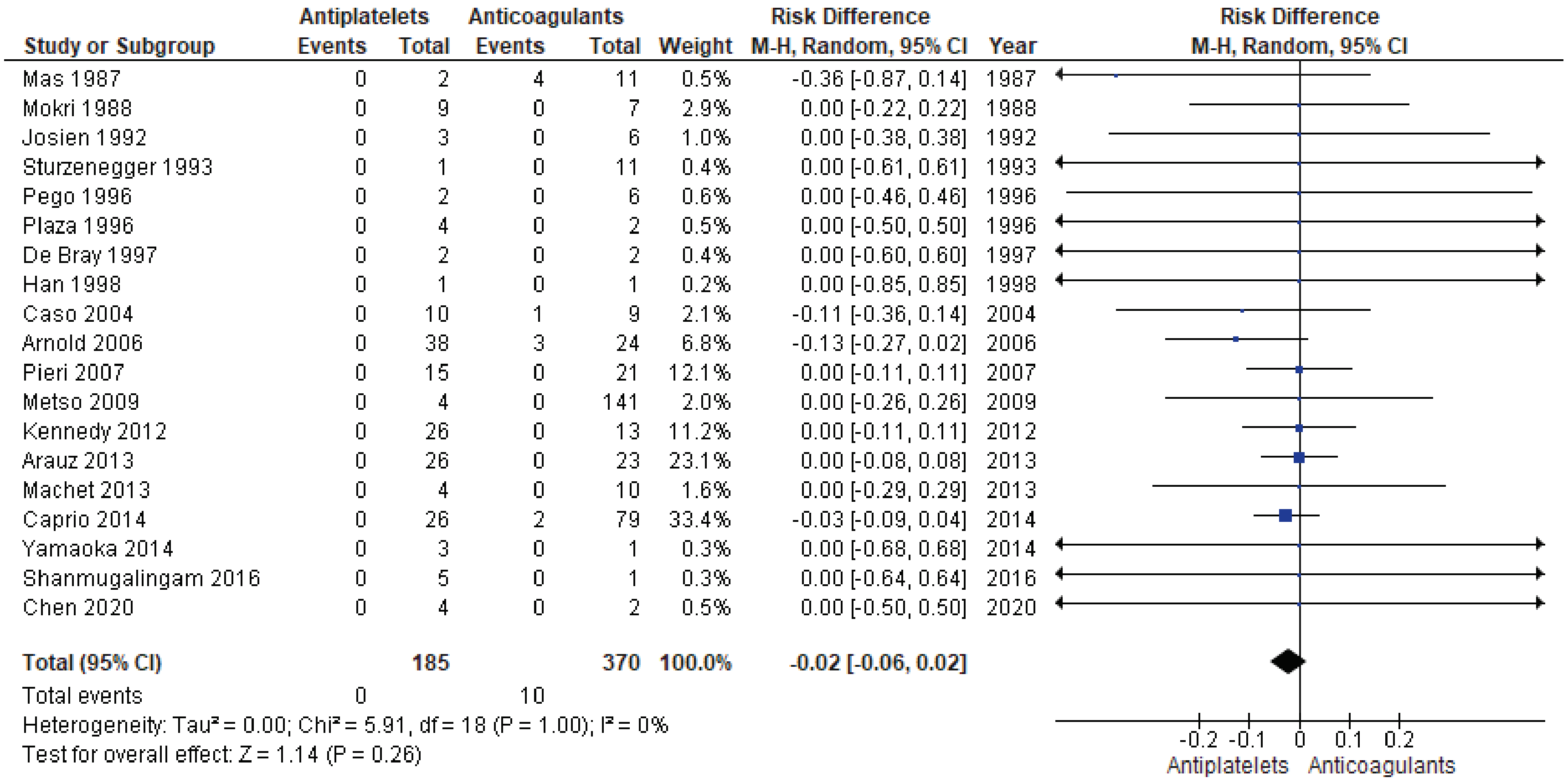

Figure S5. Forest plot for ischaemic stroke or TIA outcome in vertebral artery dissection alone

	Antiplatelets		Anticoagulants			Risk Difference	
Study or Subgroup	Events	Total	Events	Total	Weight	M-H, Random, 95% CI	Year
Mas 1987	0	2	0	11	1.9%	0.00 [-0.44, 0.44]	1987
Mokri 1988	0	9	0	7	7.9%	0.00 [-0.22, 0.22]	1988
Josien 1992	0	3	0	6	2.6%	0.00 [-0.38, 0.38]	1992
Sturzenegger 1993	0	1	0	11	1.0%	0.00 [-0.61, 0.61]	1993
Pego 1996	0	2	0	6	1.7%	0.00 [-0.46, 0.46]	1996
Plaza 1996	0	4	0	2	1.5%	0.00 [-0.50, 0.50]	1996
De Bray 1997	0	2	0	2	1.0%	0.00 [-0.60, 0.60]	1997
Han 1998	0	1	0	1	0.5%	0.00 [-0.85, 0.85]	1998
Arnold 2006	2	38	6	24	10.5%	-0.20 [-0.38, -0.01]	2006
Pieri 2007	0	15	0	21	33.1%	0.00 [-0.11, 0.11]	2007
Kennedy 2012	0	26	0	13	30.6%	0.00 [-0.11, 0.11]	2012
Machet 2013	0	4	0	10	4.4%	0.00 [-0.29, 0.29]	2013
Yamaoka 2014	0	3	0	1	0.8%	0.00 [-0.68, 0.68]	2014
Shanmugalingam 2016	0	5	0	1	0.9%	0.00 [-0.64, 0.64]	2016
Chen 2020	0	4	0	2	1.5%	0.00 [-0.50, 0.50]	2020
**Total (95% CI)**		**119**		**118**	**100.0%**	**-0.02 [-0.08, 0.04]**	
Total events	2		6				

Heterogeneity: Tau² = 0.00; Chi² = 5.20, df = 14 (P = 0.98); I² = 0%
Test for overall effect: Z = 0.67 (P = 0.50)

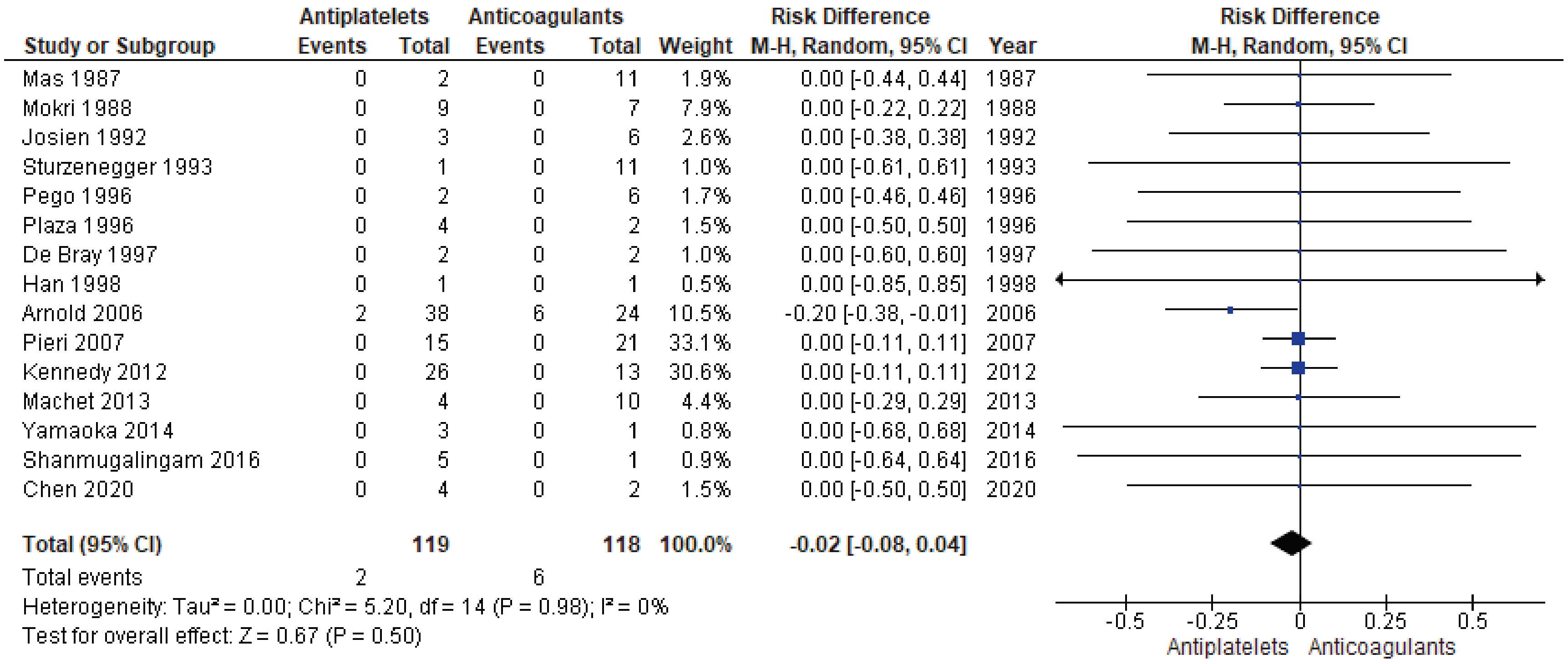

Figure S6. Forest plot for death outcome in vertebral artery dissection alone

Study or Subgroup	Antiplatelets Events	Antiplatelets Total	Anticoagulants Events	Anticoagulants Total	Weight	Risk Difference M-H, Random, 95% CI	Year
Mas 1987	0	2	0	11	1.1%	0.00 [-0.44, 0.44]	1987
Mokri 1988	0	9	0	7	4.3%	0.00 [-0.22, 0.22]	1988
Josien 1992	0	3	0	6	1.4%	0.00 [-0.38, 0.38]	1992
Sturzenegger 1993	0	1	0	11	0.5%	0.00 [-0.61, 0.61]	1993
Pego 1996	0	2	1	6	0.7%	-0.17 [-0.69, 0.35]	1996
Plaza 1996	0	4	0	2	0.8%	0.00 [-0.50, 0.50]	1996
De Bray 1997	0	2	0	2	0.6%	0.00 [-0.60, 0.60]	1997
Han 1998	0	1	0	1	0.3%	0.00 [-0.85, 0.85]	1998
Gonzales-Portillo 2002	0	4	0	4	1.5%	0.00 [-0.37, 0.37]	2002
Caso 2004	0	10	1	9	3.1%	-0.11 [-0.36, 0.14]	2004
Arnold 2006	2	38	0	24	22.6%	0.05 [-0.04, 0.15]	2006
Metso 2009	0	4	1	141	2.9%	-0.01 [-0.27, 0.26]	2009
Kennedy 2012	0	26	0	13	16.7%	0.00 [-0.11, 0.11]	2012
Arauz 2013	0	26	0	23	34.4%	0.00 [-0.08, 0.08]	2013
Machet 2013	0	4	0	10	2.4%	0.00 [-0.29, 0.29]	2013
Yamaoka 2014	0	3	0	1	0.4%	0.00 [-0.68, 0.68]	2014
Gensicke 2015	0	20	0	6	5.0%	0.00 [-0.20, 0.20]	2015
Shanmugalingam 2016	0	5	0	1	0.5%	0.00 [-0.64, 0.64]	2016
Chen 2020	0	4	0	2	0.8%	0.00 [-0.50, 0.50]	2020
**Total (95% CI)**		**168**		**280**	**100.0%**	**0.01 [-0.04, 0.05]**	
Total events	2		3				

Heterogeneity: Tau² = 0.00; Chi² = 2.34, df = 18 (P = 1.00); I² = 0%
Test for overall effect: Z = 0.30 (P = 0.76)

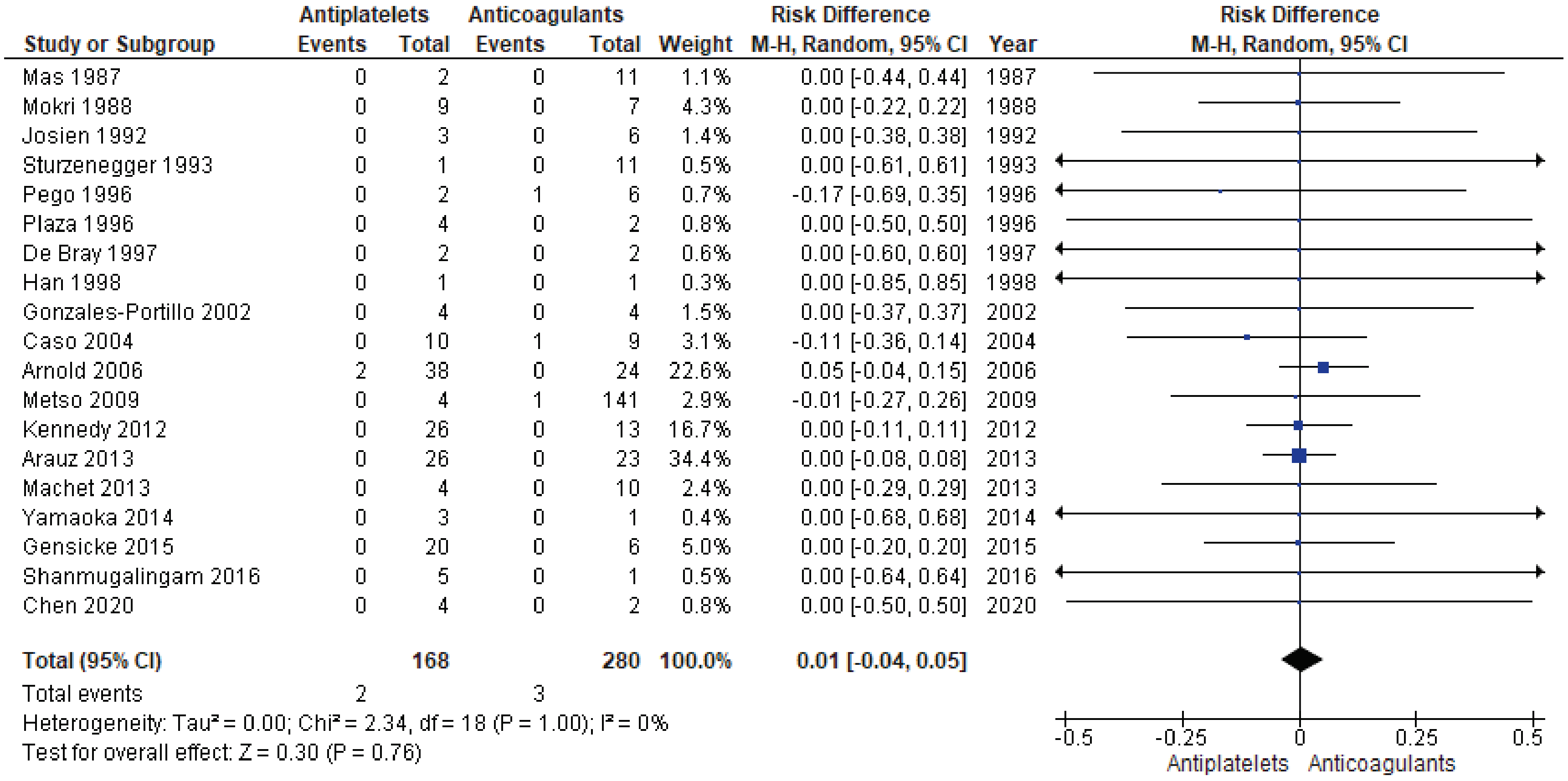

Supplement: Supplementary file 1 — Additional file1: Table S1. Search words used and the steps involved for MEDLINE database search. Figure S1. Forest plot for ischaemic stroke outcome in carotid artery dissection alone. Figure S2. Forest plot for ischaemic stroke or TIA outcome in carotid artery dissection alone. Figure S3. Forest plot for death outcome in carotid artery dissection alone. Figure S4. Forest plot for ischaemic stroke outcome in vertebral artery dissection alone. Figure S5. Forest plot for ischaemic stroke or TIA outcome in vertebral artery dissection alone. Figure S6. Forest plot for death outcome in vertebral artery dissection alone. [file 42466_2022_188_MOESM1_ESM.pdf]
